# A Linear Epitope in the N-Terminal Domain of CCR5 and Its Interaction with Antibody

**DOI:** 10.1371/journal.pone.0128381

**Published:** 2015-06-01

**Authors:** Benny Chain, Jack Arnold, Samia Akthar, Michael Brandt, David Davis, Mahdad Noursadeghi, Thabo Lapp, Changhua Ji, Surya Sankuratri, Yanjing Zhang, Lata Govada, Emmanuel Saridakis, Naomi Chayen

**Affiliations:** 1 Division of Infection and Immunity, UCL, Gower St., London, United Kingdom; 2 Virology Discovery and Translational Area, Roche Nutley, 340 Kingsland Street Nutley, NJ 07110, United States of America; 3 Department of Virology, Biomedical Primate Research Centre, Rijswijk, The Netherlands; 4 Computational and Systems Medicine, Department of Surgery and Cancer, Faculty of Medicine, Imperial College London, London, United Kingdom; 5 Laboratory of Structural and Supramolecular Chemistry, Department of Physical Chemistry, National Centre for Scientific Research 'Demokritos', Athens, Greece; National Cancer Institute, NIH, UNITED STATES

## Abstract

The CCR5 receptor plays a role in several key physiological and pathological processes and is an important therapeutic target. Inhibition of the CCR5 axis by passive or active immunisation offers one very selective strategy for intervention. In this study we define a new linear epitope within the extracellular domain of CCR5 recognised by two independently produced monoclonal antibodies. A short peptide encoding the linear epitope can induce antibodies which recognise the intact receptor when administered colinear with a tetanus toxoid helper T cell epitope. The monoclonal antibody RoAb 13 is shown to bind to both cells and peptide with moderate to high affinity (6x10^8 and 1.2x10^7^ M^-1^ respectively), and binding to the peptide is enhanced by sulfation of tyrosines at positions 10 and 14. RoAb13, which has previously been shown to block HIV infection, also blocks migration of monocytes in response to CCR5 binding chemokines and to inflammatory macrophage conditioned medium. A Fab fragment of RoAb13 has been crystallised and a structure of the antibody is reported to 2.1 angstrom resolution.

## Introduction

The chemokine receptor CCR5 and its ligands CCL3 (MIP1α), CCL4 (MIP1β) and CCL5 (RANTES) play an important role in orchestrating the inflammatory response [1]. CCR5 mediated inflammation may play an important role in promoting the growth of tumours[2] and in other diseases exhibiting chronic inflammatory pathologies [3]. The CCR5 is also one of the main entry co-receptors for HIV, and CCR5 deficiency is strongly linked to protection against infection[4,5]. Furthermore, an individual who received a stem cell transplant from a CCR5 negative donor (for treatment of acute myeloid leukemia) is believed to be the only patient to have been cured of HIV [6]. For all these reasons, there has been and continues to be great interest in blocking CCR5 function. One approach to this goal is the development of antibodies as functional inhibitors of CCR5, since antibodies can provide high effectiveness coupled with very low toxicity [7].

CCR5 has also been considered as a potential target for (auto) vaccination, by inhibiting binding of ligands or to induce downregulation of the receptor from the cell surface. Vaccines against CCR5 avoid the problem of virus variability and viral escape. Several groups have investigated the possibility of raising antibodies against CCR5[8–13], and have used recombinant proteins, recombinant viruses or synthetic cyclic peptides to provide proof of principal evidence that the strategy can work. The safety of autoantigen driven vaccine strategies remains a cause for concern, however. A trial of therapeutic vaccination in Alzheimer patients using the amyloid fragment Aβ, was discontinued because of adverse side effects attributed to the autoimmune response [14], although the damage may have been due to autoimmune cellular rather than humoral responses. Cellular autoimmune responses against the CCR5 receptor are likely to be pathogenic, since they may lead to elimination of dendritic cells, macrophages, T cells and any other cell types which express this receptor. We have previously explored the possibility of raising an immune response to the CCR5 receptor[15], using a very short N-terminal fragment of the receptor, coupled to a well characterised epitope of tetanus toxoid [16,17]. Since the immunogen contained only a short stretch of CCR5 sequence, the possibility of including a CD4 or CD8 T cell auto-epitope is minimised. Furthermore, since tolerance is mediated primarily at the level of T cells (whether via deletion or regulatory T cells), and T cell help in this model is provided by a non-self epitope, the strategy should help overcome auto-tolerance to CCR5. However, our previous studies demonstrated that only a small proportion of the antibody response against the N-terminal seven amino acids of CCR5 reacted with the intact receptor on the surface of cells.

In this study we have examined a number of available monoclonal antibodies raised against intact human CCR5, and identified two which recognise a synthetic peptide spanning the N-terminal domain of CCR5. Both antibodies (deriving from completely independent immunizations in different laboratories) were found to target the same core stretch of amino acids. We then synthesised a synthetic peptide coding this minimal epitope co-linear with a tetanus toxoid sequence coding for a T helper epitope and used this chimeric peptide to stimulate an antibody response in mice, and showed that serum from the peptide immunised mice recognised surface CCR5. Having characterised the peptide epitope recognised by the monoclonal antibodies, we further characterised the functional and structural characteristics of one of the monoclonal antibodies recognising the linear epitope. This antibody was sequenced, and then its Fab fragment was crystallised and the structure solved at high resolution. The study provides the first characterization of a linear epitope within the CCR5 protein, together with its cognate antibody ligand.

## Materials and Methods

### Antibodies

Purified mouse monoclonal antibodies180 (clone 45502, Ig2b), 1801 (Clone CTC8, IgG1) and 1802 (Clone CTC5, IgG1)were obtained from R&D Systems (R&D Systems Europe Ltd., Abingdon, OX14 3NB, UK). All three were raised by immunising mice with CCR5 expressing transfectants as described in [18]. The RoAb13 hybridoma (line number PZ3733) was provided by Roche Palo Alto LLC. Female Balb/c mice were given a primary intraperitoneal immunization with 107 CCR5-expressing cells (CHO-CCR5 or L1.2-CCR5) with complete Freund’s adjuvant. The second immunization was done 4–6 weeks later similarly except incomplete Freund’s adjuvant was used with the cells. The mice were then boosted at 4–6 week intervals with 10^7^ CHO-CCR5or L1.2-

CCR5cells in phosphate-buffered saline (PBS) with no adjuvant. The last immunization was carried out intraperitoneally with 10^7^ CCR5-expressing cells on the third day before fusion. The spleen cells of the immunized mice were fused with myeloma cells. Ten days after fusion, the supernatants were tested for specific antibody production by cell-based ELISA. Hybridoma ROAb13 that produced the anti-CCR5 mAb clone ROAb13 was cloned by limiting dilution[19].

The hybridoma was expanded inHyclone ADCF Mab Medium with L-Glutamine (Fisher Scientific, #SH3034901) supplemented with Nutridoma-SP (Roche, #11011375001) as per manufacturer’s instructions. The antibody supernatant was purified by two sequential affinity purifications on Protein-A Sepharose (Sigma). The column was washed with > 10 column volumes of 0.15M NaCl 20mM Tris buffer, pH 8.0, and the antibody was eluted with 0.1M Citrate Buffer, pH 3.5, and dialysed against PBS. Purified antibody was stored in batches at -80°C. Fab fragment was produced by incubating the antibody with Papain (2.5 μg/ml, Sigma, #P3125) in the presence of 1mM EDTA, 5 mM Cysteine for 4 hours at RT. The reaction was stopped by addition of iodoacetamide (1 μg/ml) and incubation for 15 minutes at RT. The Fc fragment was removed by passage over the protein A column again, and the flow through was dialysed against 0.1M NaCl, 0.05 M Hepes, pH 7 and concentrated by filtration using Centriprep YM-10,10 kDa NMWL (Millipore). The purity and yield of intact antibody and Fab was assessed by PAGE electrophoresis (see Supporting Information S4 Fig).

### Peptides

The peptide encoding the N-terminal extracellular domain of human CCR5 (MDYQVSSPIYDINYYTSEPCQKINVKQIAAIC-biotin, hCCR5_1–31_) was synthesised by the Peptide Synthesis Laboratory, Cancer ResearchUK. The same peptide without the C-terminal cysteine biotin was synthesised by PeptideSynthetics (Fareham PO15 6DP). An analogue of the peptide with sulphated tyrosines at position 10 and 14 was synthesised as described previously [20] and given as a gift by Prof. Fred Naider (City University of New York). The panel of truncated peptides used for epitope mapping (See Results, Subsection “Defining a linear epitope in the N-terminal domain of CCR5”) was synthesised by Sigma (Pepscreen). The chimeric peptide used for immunisation (SPIYDINYYTGPSLVDDALINSTKIYSYFPSV) was synthesised by the peptide synthesis facility at the University of Ghent. All peptides were >80% pure by HPLC and mass spectroscopy.

### CCR5 transfectants

Chinese hamster ovary cells were transfected by electroporation with vectors expressing human or mouse CCR5 in an expression vector containing a neomycin resistance gene (Genecopoeia, #EX-Z0659-M02 and #EX-Mm01826-M02 respectively). G418 resistant cells were cloned by limit dilution and receptor expressing cells were identified by flow cytometry using RoAb13 anti-CCR5 and Rabbit anti-mouse FITC for human CCR5, and PE anti-Mouse CD195 (CCR5) (clone HM-CCR5, Insight Biotechnology) for mouse CCR5.

### ELISA

96 well ELISA plates (Nunc) were coated with peptide conjugates (10 μg/ml unless otherwise stated) in coating buffer (0.1 M sodium bicarbonate, pH 8.5), overnight at 4°C. All wells were washed (washing buffer PBS 0.1% Tween-20) and blocked by addition of 2% low fat milk (Tesco) in PBS for at least 1 h at room temperature. In some experiments biotinylated peptide was mixed with avidin (in 4:1 peptide to avidin ratio) for 15 minutes before coating on the plate, as this was found to increase binding. Unconjugated avidin was used as control coating. Sera or antibodies were diluted in washing buffer and added at the concentrations shown, for 1.5–3 h at room temperature. Bound antibody was detected using alkaline phosphatase coupled rabbit anti-mouse IgG or IgGFab (Sigma) antibody (1:2000 dilution) and substrate (PNPP FAST, Sigma). Results are shown as optical density (405 nm).

For measurement of affinity, antibody (purified Fab fragment) at different dilutions was mixed with a fixed concentration of hCCR5_1–31_ and incubated for three hours or overnight at 4°C to reach equilibrium. The amount of “free” antibody was then measured by using a standard ELISA as described above, and comparing to a standard curve of different concentrations of purified RoAb13. Since total amount of antibody in each well was known, the bound could be calculated as total-free. Each concentration was tested in triplicate. A two-stage ELISA was used in preference to a single-stage ELISA for these quantitative assays, in order to avoid the avidity effects of peptide presented on a plastic surface interacting with divalent antibody.

The pepscreen panel of CCR5 truncated peptides was screened by competitive ELISA. Antibody supernatant (typically diluted 1 in 1000 or 1 in 4000) was added to N-terminal domain peptide coated ELISA plates in the presence or absence of 1 μg/well of one of the competing peptides. The antibody peptide mixture was incubated overnight, washed and then bound antibody measured as described above.

### Flow cytometry

CHO cell transfectants (5 × 10^4^) were incubated in 10% goat serum (Sigma) and 0.1% sodium azide for 30 min at 4°C. Purified antibodies or mouse sera at various dilutions were added, and incubated for 2–3 h at 4°C. Cells were washed and bound antibody detected using goat anti-rabbit FITC or APC conjugates (R&D Systems). The number of bound antibody molecules was quantified in some experiments using Quikfit calibration beads containing known numbers of bound mouse IgG molecules as per manufacturers instructions (DAKO, #K0078).

Peripheral blood subpopulations were stained using purified RoAb13 Fab fragment directly conjugated with Alexa 482 as per manufacturer’s instructions.

Peripheral blood mononuclear cells (PBMCs) derived from a rhesus macaque (*Macacamulatta*) were used in this study. The macaque was captive bred for research purposes and socially housed at the BPRC. BPRC facilities comply with Dutch law on animal experiments (Wet op de Dierproeven and its adaptations as published in the Staatscourant), the European Council Directive 86/609/EEC, as well as with the ‘Standard for humane care and use of Laboratory Animals by Foreign institutions’ identification number A5539-01, provided by the Department of Health and Human Services of the United States of America’s National Institutes of Health (NIH). Enrichment was provided in the form of pieces of wood, mirrors, food puzzles, a variety of food and other home made or commercially available enrichment products. Animals are fed with standard food pellets, fruit and bread. Water is provided *ad libitum*.

Animals are monitored daily for health and discomfort. The Institutional Animal Care and Use Committee (BPRC DierExperimentenCommissie, DEC) pre-approved all procedures. The qualification of the members of this committee, including their independence from a research institute, is requested in the Wet op de Dierproeven (1996). At the BPRC all animal handling is performed within the Department of Animal Science (ASD) according to Dutch law. A large experienced staff is available including full time veterinarians and a pathologist. The ASD is regularly inspected by the responsible authority (VoedselenWarenAutoriteit, VWA) and an independent Animal Welfare Officer. All steps are taken to ameliorate the welfare and to avoid any suffering of the animals. The macaque was sedated with ketamine before blood was taken. The animal from which peripheral blood was obtained was not used exclusively for this purpose, in full accordance with the 3Rs, reducing the numbers involved in animal experiments. No animal was sacrificed during the course of these studies. The Council of the Association for Assessment and Accreditation of Laboratory Animal Care (AAALAC International) has awarded full accreditation to the BPRC. Thus, the BPRC is fully compliant with international demands on animal studies and welfare as set out by the European Convention for the Protection of Vertebrate Animals used for Experimental and other Scientific Purposes, Council of Europe (ETS 123 including the revised Appendix A), Dutch implementing legislation and the Guide for Care and Use of Laboratory Animals.

The ability of antibodies to bind to rhesus macaque CCR5 was determined by fluorescence-activated cell scanning analysis of peripheral blood mononuclear cells. For multi-color staining, 50 to 100 μl of cells were incubated with 25 μl of the monoclonal antibody for 15 min at room temperature. The cells were centrifuged for 10 min at 200 × g. Supernatant was aspirated, and the cells were resuspended in phosphate-buffered saline.

Flow cytometry was performed on a FACS ARIA using the DIVA software (Becton Dickinson). Cells were gated on the basis of forward and side scattering. At least 10,000 events were analyzed. Antibodies against CD4 conjugated to R-phycoerythrin and a cyanine dye (Cy7) (Becton Dickinson, Lincoln Park, N.Y.) and the 2D7 monoclonal antibody (Pharmigen, Woerden, The Netherlands) against CCR5 (= positive control) and conjugated to phycoerythrin-A were used. Binding of test monoclonal antibodies was detected using a goat anti-rhesus mouse antibody conjugated to phycoerythrin-A.

### Immunisation

Outbred ICRmice were purchased from Harlan and maintained in the Biological Services unit at UCL. Mice were immunised with 50 μg peptide emulsion in complete Freunds adjuvant injected subcutaneously at base of tail. The mice were boosted intraperitoneally with the same amount of peptide in Incomplete Freunds Adjuvant at weeks 2, 6 and 10 weeks. Sera were collected after a further 2–4 weeks.

### Transwell migration assay

Peripheral blood mononuclear cells and monocyte derived macrophages were prepared from peripheral blood as previously described [21]. The study was approved by the joint University College London / University College London Hospitals NHS Trust Human Research Ethics Committee and written informed consent was obtained from all participants.

Macrophages were allowed to differentiate for six days, the medium was changed to RPMI and 10% foetal calf serum (FCS) (Biosera) with or without 100 ng/mL LPS for 6 hours or 10ng/mL IFNg for 24 hours, using 1 mL media /10^6^ MDM. Conditioned media from these cultures were then centrifuged at 10,000g for 5 minutes and stored at -80°C.

Polycarbonate transwell inserts (Corning No. 3421, with 5.0 mm pores and 6.5 mm diameter) were placed over 500 μL conditioned media or control media or media containing purified chemokines in 24-well plates. 100 mL of freshly isolated PBMC (5x10^6^ cells /mL) were loaded into the upper chamber of the transwell, and incubated for 3 hours at 37°C. Transwells were then lifted out of the lower chamber and the contents of the upper chamber were carefully aspirated. Each transwell filter was subsequently tilted to rinse the under surface twice with 200 μL 0.25% Trypsin-EDTA (Gibco) that was allowed to drain into the well (lower chamber). Transwells were lowered into the bottom chamber again and incubated at 37°C for further 10 minutes to detach any cells still adherent to the under surface of the transwell filter. The lower chamber cell suspension was collected and fixed with 1% paraformaldehyde in phosphate buffered saline (PBS). Cells were then centrifuged at 400g for 5 minutes and resuspended in 100μL PBS with 0.5% bovine serum albumin (Sigma) and 0.02% sodium azide (Sigma). Cells were stained with a directly conjugated antibody to CD14 (Becton Dickinson, clone M5E2) and 10μl Flow-Check polystyrolfluorospheres (Beckman Coulter) were added for flow cytometric quantitation of monocytes identified by light scatter properties and positive staining for CD14. A FACScalibur flow cytometer (Becton Dickinson) was used with FlowJo (version 9.4.3) analysis software. 10 000 beads were counted to standardised cell count quantitation in each sample and transmigration of cells in the lower chamber was expressed as the proportion of the input.

### Sequencing of RoAb13

The heavy and light chains of RoAb13 were amplified from the hybridoma cDNA, by rapid amplification of cDNA ends (5’ RACE). Briefly, the RNA from the hybridoma was reverse transcribed using constant region primers. The 5’ end of the transcript was extended by addition of adenosines using terminal deoxynucleotidyltransferase, and the resulting molecules were amplified using a poly T primer and 3’ constant region primers ([22][23]. The primers used are given in Supporting Information S1 Table. The PCR amplicons were cloned into T-vector (Life Technologies) and sequenced by Sanger sequencing at the UCL Genomics facility. The sequences have been submitted to EMBL with accession numbers LN832627 and LN832628.

### Crystallisation of RoAb13

A solution of RoAb13 in 20 mM HEPES pH 7.0 and 0.1M NaCl was screened with a variety of commercial crystallisation screens (purchased from Hampton Research, USA, Qiagen, Molecular Dimensions, UK) in order to find suitable crystallisation conditions. Crystals appeared in 20% PEG 4K, 0.005M Nickel chloride, 0.1M Tris pH 8.5 and at pH 7.5.

Further trials were carried out by setting up 2 μl drops using the vapour diffusion in EasyXtal Tools from Qiagen and microbatch[24] in 72 well HLA plates from Douglas Instruments at 20°C. Vapour diffusion set ups were performed by mixing equal volumes of the Fab fragment and crystallisation solutions on the screw cap of the Easy Xtal plate and inverted over a 300μl crystallisation solution in the reservoir. In the case of microbatch, trials were incubated under a layer of paraffin oil (VWR International). All other reagents and chemicals were obtained from Sigma- Aldrich, UK.

Optimisation of the above conditions were performed with 20% (w/v) PEGs of different molecular weights including 600, 1000, 1500, 2000MME, 3350, 5000MME, 8000, 10 and 20,000 Da. The concentrations of Nickel chloride was varied between 0.005Mand 0.01 M in steps of 0.001M. The 0.1M Tris buffer ranged between pH 7.5 to 8.5 in increments of 0.2.

The best crystals diffracting to 2.1 Å were obtained in the vapour diffusion experiments with 20% (w/v) PEG2K MME, 0.01M Nickel Chloride and 0.1M Tris pH 7.5 after 7 days.

### Data collection and structure determination

Data were collected from a single crystal at 100°K using a RigakuMicromax 007HF-M high-flux X-ray generator equipped with Osmic VariMax optics, a Rigaku Saturn 944+ CCD detector and an Oxford Cryosystems 700 cryostream, at the X-ray Crystallography Facility of the Centre for Structural Biology, Imperial College London. They were processed to 2.10 Å resolution using the Mosflm/Scala software [25][26]. The crystal belonged to space group P2_1_ with unit cell dimensions a = 75.20 Å, b = 88.64 Å, c = 78.45 Å and beta = 103.19°. The structure was solved by molecular replacement using Molrep[27] with the structure of PDB ID no. 1HIL as a search model, and refined, initially with the Refmac 5 maximum likelihood refinement program [28] and then with alternating cycles of least-squares refinement using Phenix.refine[29] and manual fitting with Coot [30], reserving 5% of the diffraction intensities for R_free_ calculation. The structure was refined to R and R_free_ values of 18.6% and 23.4% respectively, and consisted of 6726 non-hydrogen protein atoms and 789 water molecules. There were two dimers in the asymmetric unit (labeled H/L and A/B). The H chain (A in the second dimer) consists of 224 residues and the L chain (B in the second dimer) of 217. There was no assignable electron density corresponding to residues 135–138 of the H and A chains and these residues are therefore omitted from the coordinates file. All atoms, including solvent, were refined with an occupancy of 1, except for the side-chains of residues Gln 5, Arg 44, Ser 62, Arg 102 and Gln 111 of chain H, Arg 161 of chain L, and Ser 25 and Arg 161 of chain B, which were each refined with two alternative conformations. Data collection and refinement statistics are summarised in Supporting Information S2 Table. The coordinates have been deposited at PDB (PDB ID code 4S2S).

## Results

### Defining a linear epitope in the N-terminal domain of CCR5

**Fig 1:**
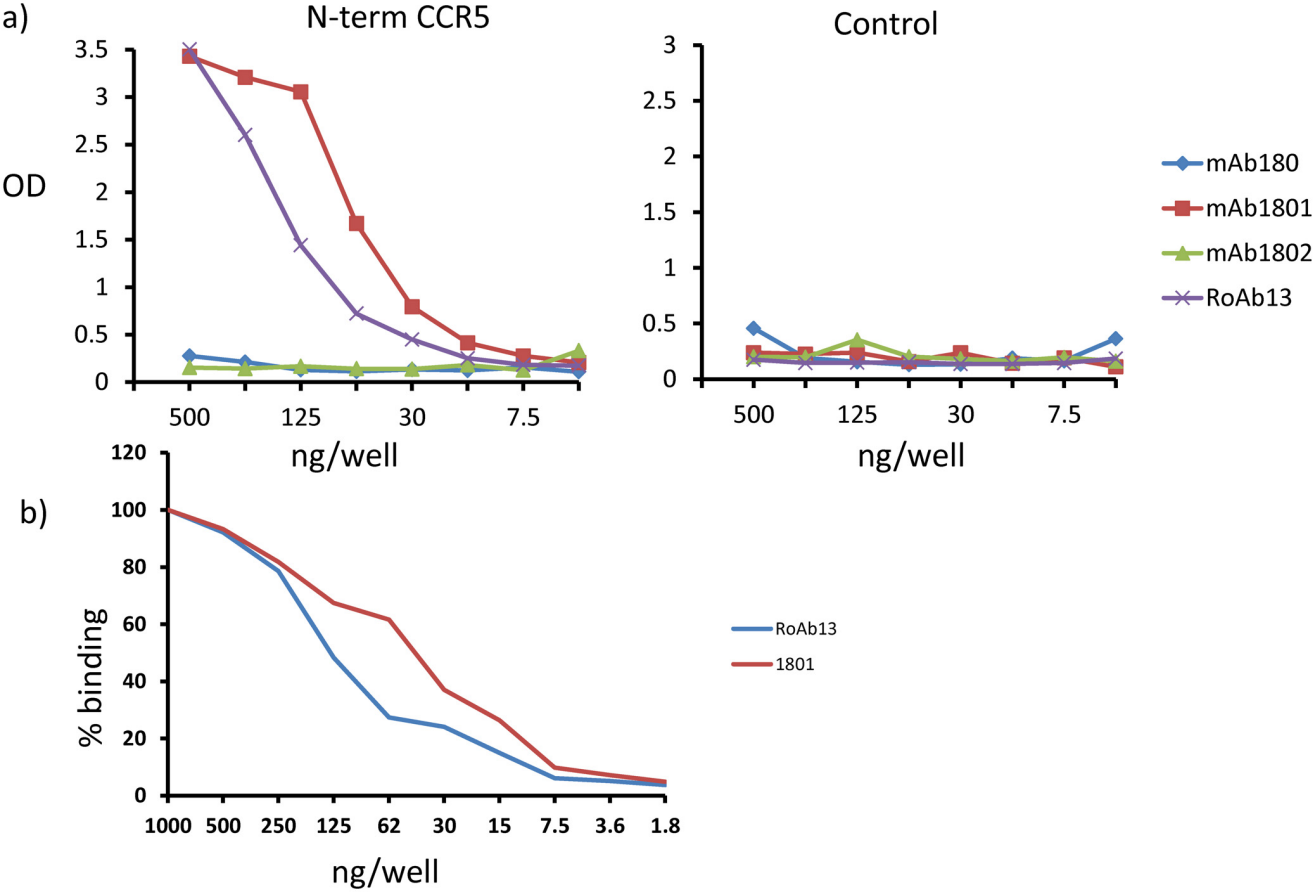
Two monoclonal antibodies recognize a linear epitope in the N-terminal domain of CCR5. A, Dilutions of the four monoclonal antibodies shown were tested for binding to hCCR5_1–31_coding for the N-terminal extracellular domain of CCR5 by ELISA as described in M&M. The left panel shows binding (as measured by OD at 405 nm) to plates coated with the N-terminal peptide coupled to avidin, while the right panel shows binding to avidin alone. B, Dilutions of R&D1801 and RoAb13 were tested for binding to CCR5-transfected CHO cells by indirect immunofluorescence and flow cytometry. The results are normalized and expressed as % maximum binding for each antibody. Binding to untransfected controls or to CHO cells transfected with mouse CCR5 was less than 5% of maximum binding in each case.

We tested four antibodies which were isolated from mice immunised with cells over expressing CCR5. All four antibodies recognised native cell surface CCR5 [18,19]. All four have been reported to recognise CCR5 in Western Blot, and hence were believed to target linear, rather than conformational epitopes of CCR5. Three of the antibodies were available commercially as purified IgG (R&D Systems), while the fourth RoAb13 was produced and made available for this study by Roche Palo Alto. The antibodies were tested in ELISA against a synthetic peptide coding the entire N-terminal domain of human CCR5 (hCCR5_1–31_) (Fig 1a). Two antibodies, R&D1801 and RoAb13 recognised the synthetic peptide, confirming that the antibodies were able to recognise a linear epitope in the first extracellular domain of CCR5. As reported previously, both antibodies also bound strongly to CCR5-expressing transfectants (Fig 1b).

**Fig 2:**
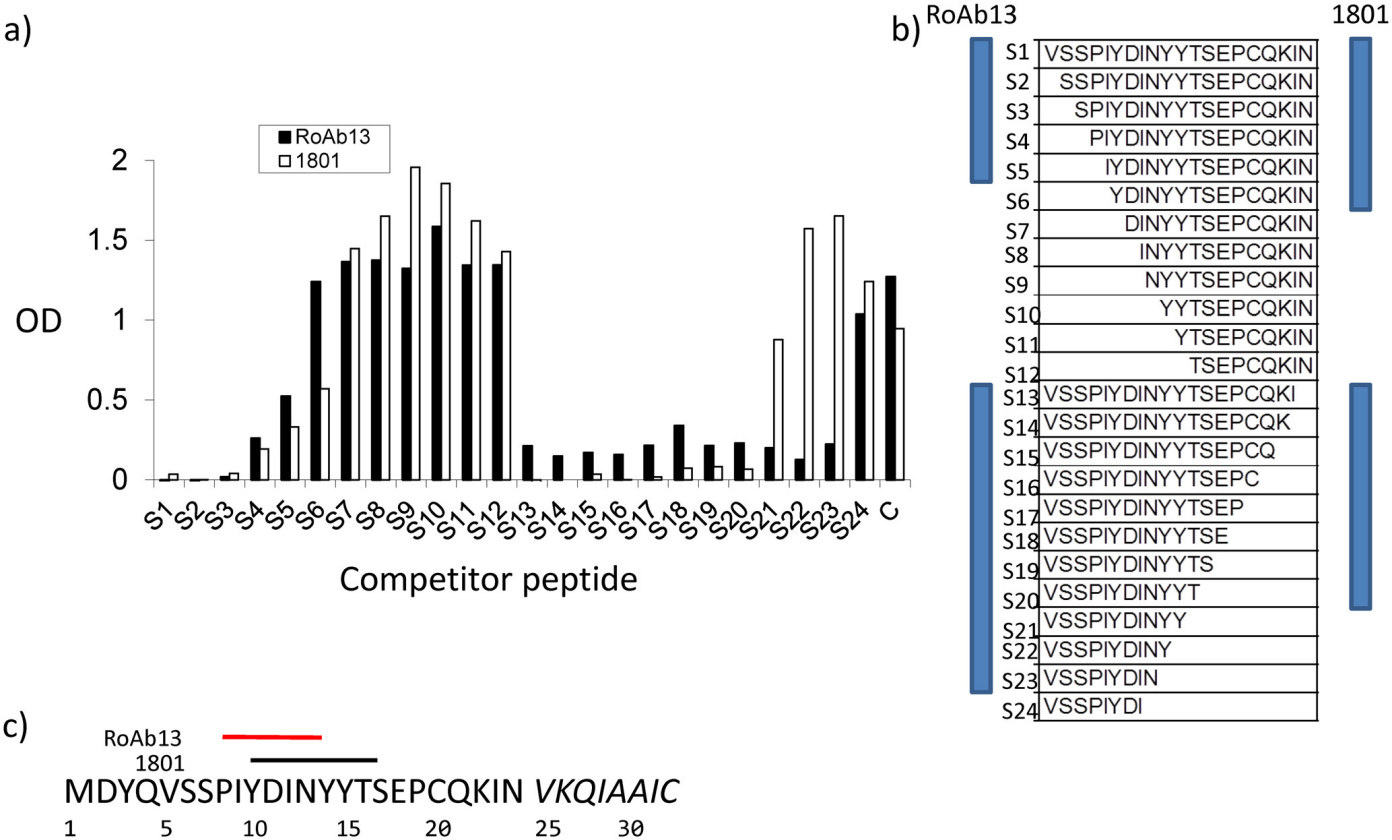
R&D1801 and RoAb13 recognise overlapping core epitopes in the CCR5 N-terminal domain. A, R&D1801 or RoAb13 were incubated overnight with competing peptides 1–24 (1 μg/ml) in wells coated with hCCR5_1–31_, and binding (shown as OD 405 nm) of antibody was then measured as in Fig 1a using a rabbit anti-mouse second layer. C—PBS used in place of competitive peptide. B, The peptide sequence of the 24 peptides tested in a. The blue bars shows all peptides which inhibited the binding by more than 50%. C, Diagrammatic representation of the core binding epitope recognized by the two antibodies, defined by peptides which inhibited binding to the whole N-terminal peptide by more than 50%.

We next defined the core epitope recognised by the R&D1801 and RoAb13 using a panel of truncated peptides spanning the region 5–25 (the N-terminal region of CCR5 up to the first transmembrane segment) in a competitive ELISA (Fig 2). R&D1801 binding to hCCR5_1–31_was completely blocked by a peptide spanning the whole of 5–25. Progressive truncation of the peptide at the C-terminal terminus resulted in loss of inhibition as soon as the target peptide lost the C-terminal threonine at position 16 (Fig 2a). Progressive truncation at the N-terminal terminus gave a less clear cut result, with inhibitory activity falling gradually after removal of the serine at position 7. All inhibitory activity was lost after removal of the tyrosine at position 10. The core target of this antibody is therefore contained in the 7amino acids spanning positions 10–16 (Fig 2b and 2c). RoAb13 recognised an overlapping core epitope between aa8 (proline) and 13(asparagine) (Fig 2c). The CCR5 sequence 8 to 16 therefore represents a linear epitope recognised by two monoclonal antibodies, which also recognise CCR5 expressed at the cell surface. Since we had access to the hybridoma producing RoAb13 we focused further studies predominantly on this antibody.

### Affinity of RoAb13 for linear and native forms of CCR5

**Fig 3:**
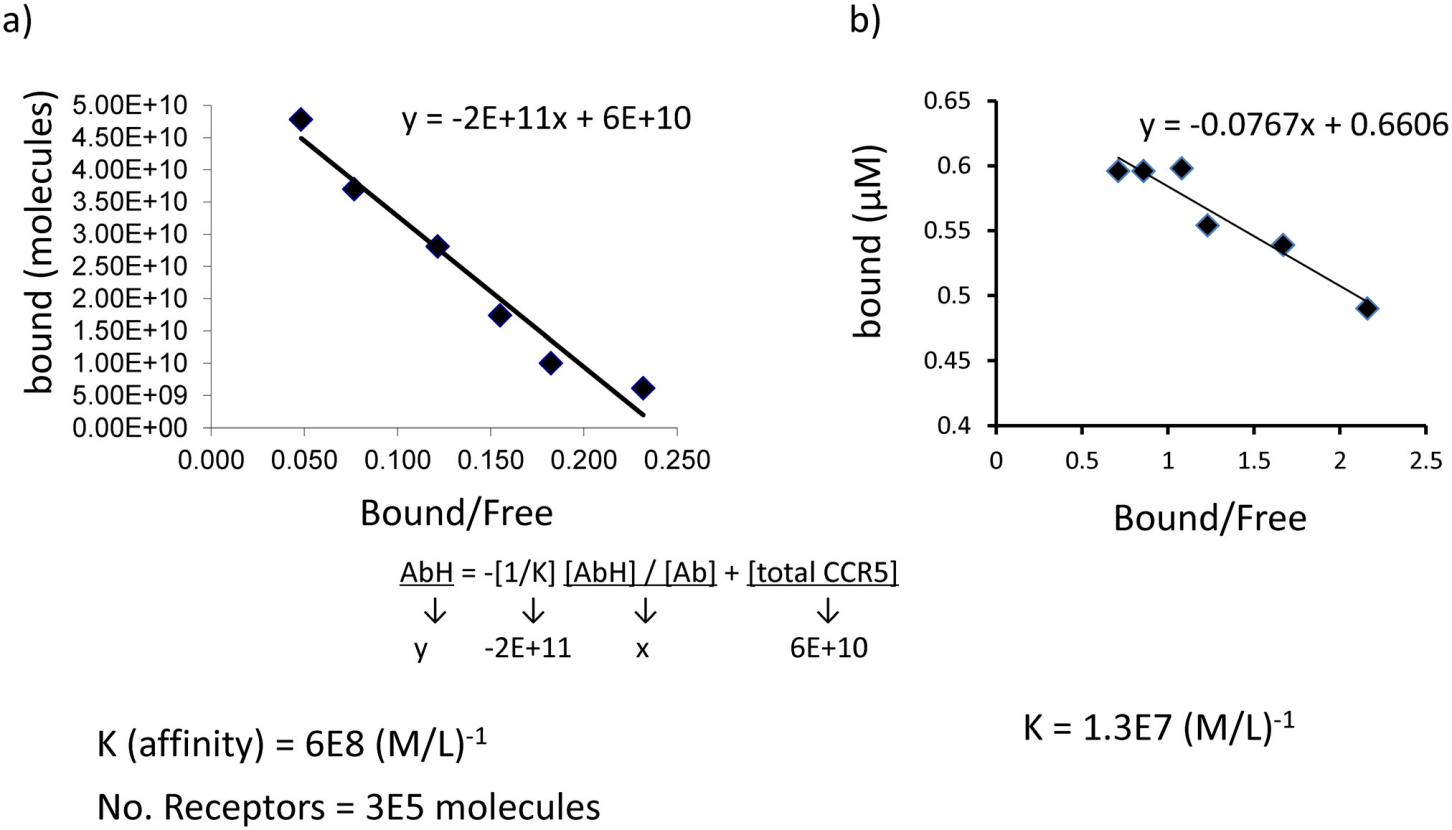
Affinity of RoAb13 binding to native cell-surface CCR5 and a peptide coding for the N-terminal extracellular domain of CCR5. A, RoAb13 was incubated with CCR5-expressing transfected cells or controls at different concentrations and binding measured by indirect immunofluorescence and flow cytometry. The binding was converted to absolute number of bound antibody molecules by using Ig calibration beads as described in M&M and shown in Supporting information S1 Fig. The graph shows the concentration of bound antibody molecules plotted against the ratio of bound/free antibody (a classical Scatchard plot). The equation showing the relationship between bound and bound/free derived from the law of mass action is shown below the figure, together with the affinity and number of receptors calculated respectively from the slope and intercept of the equation. B, RoAb13 was incubated with different concentrations of hCCR5_1–31_N-terminal domain peptide overnight. The remaining amount of free antibody was estimated by binding to hCCR5_1–31_ in ELISA and comparison to a standard curve. Bound antibody was calculated as total—free. The resulting bound and bound/free ratio was plotted as in a) and the affinity calculated from the slope.

We next measured the affinity of RoAb13 binding to cell surface CCR5 (expressed on CCR5 transfectants) by flow cytometry (fig 3a, and Supporting Information S1 Fig). A Scatchard plot showed a good linear fit to the binding data, with an estimated affinity K of 6 x 10^8^(M/L)^-1^. The affinity for binding of the antibody to hCCR5_1–31_peptide was estimated by competitive ELISA as K = 1.2x10^7^(M/L)^-1^ (Fig 3b).

### Immunization with a peptide chimera containing the minimal linear epitope of CCR5 N-terminal domain

**Fig 4:**
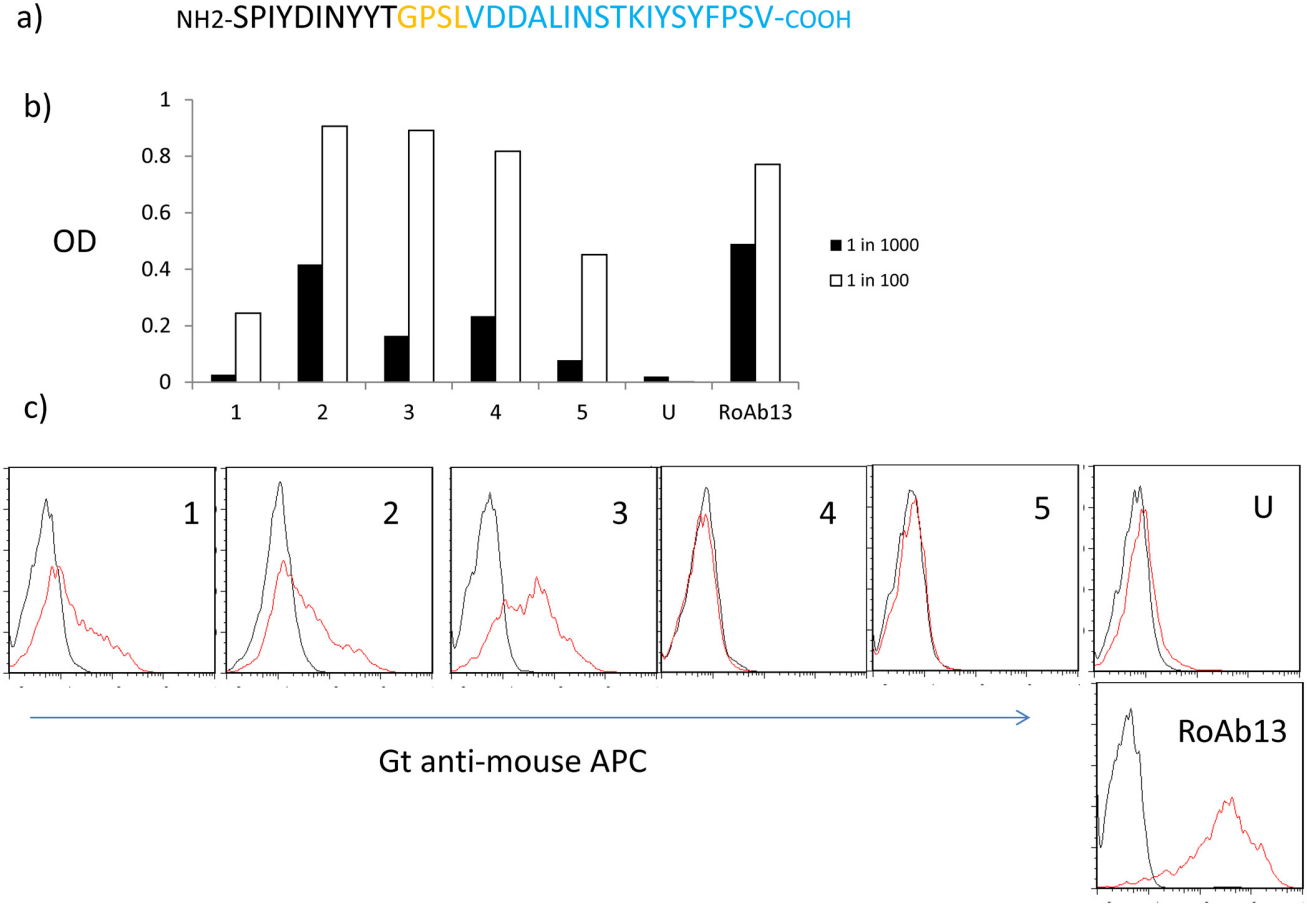
Immunisation with a chimeric peptide coding a linear CCR5 antibody epitope together with a helper epitope from tetanus toxoid can stimulate antibodies which recognize native CCR5. A, The sequence of the chimeric peptide used for immunization, showing the CCR5 B cell epitope, the linker sequence and the T cell helper epitope from tetanus toxoid. B, Sera from 5 immunized mice were collected after priming and boosting (see M&M) with the peptide shown in a), and tested in ELISA for binding to hCCR5_1–31_. Binding is shown as OD at 405 nm. U: serum from an unimmunized mouse (preimmune sera showed equivalent binding). RoAb13: supernatant from the RoAb13 hybridoma diluted as shown. C, Sera from the same five mice were tested for binding to CCR5 transfectants (red line) or controls at a dilution of 1:50.

We next tested whether the core linear epitope we defined above could be used to stimulate an antibody response which recognises native CCR5. We used the strategy adopted previously[15], in which the short linear epitope was linked to a tetanus toxoid helper epitope via a four amino acid linker (Fig 4a). We used a chimeric peptide so as to introduce a known strong helper epitope from Tetanus toxoid protein, in order that T cell help should not be limiting. The results of immunising 5 mice with this synthetic peptide are shown in fig 4b and 4c. All five mice showed a positive ELISA when measured against the whole N-terminal peptide, indicating that the short linear epitope is immunogenic in the context of the peptide chimera. The heterogenous magnitude of response may reflect the fact that the mice are outbred, and hence differ in MHC or other immunoregulatory polymorphic genes. The antibody response when tested on CCR5 expressing transfected cells wasmore variable. Mice 1–3 gave antibody responses which recognised cell surface CCR5 as well as peptide, while mice 4–5 failed to recognise native CCR5, despite reacting well with peptide. Even with positive antisera which did recognise native CCR5, the staining observed was always weaker than that of RoAb13 (Fig 4c).

**Fig 5:**
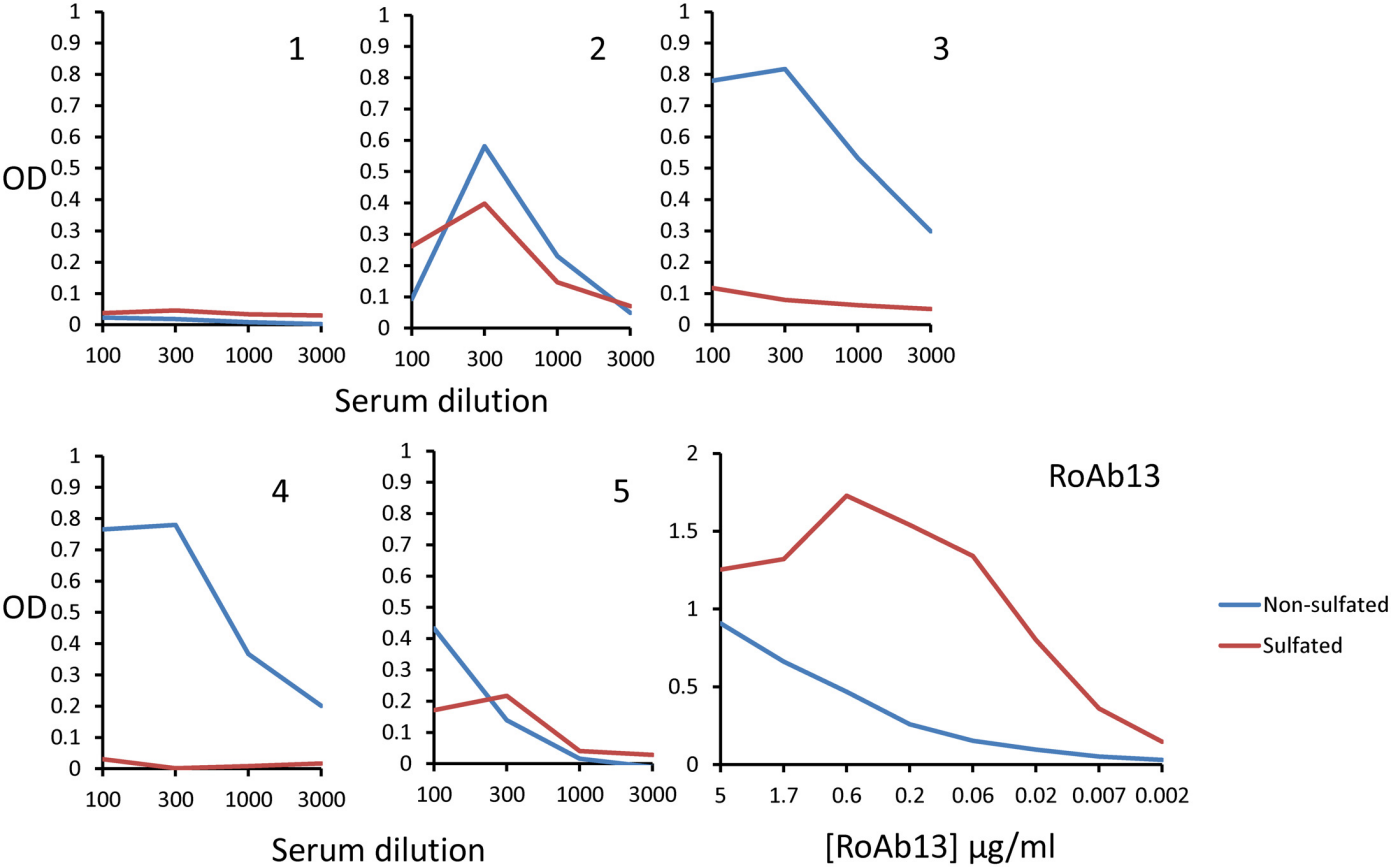
Binding of immune sera and RoAb13 to sulphated N-term CCR5. Dilutions of sera from immunized mice 1–5 (see Fig 4) or purified RoAb13 supernatant were tested by ELISA for binding to hCCR5_1–31_, or an analogue of the same peptide modified by sulfation of tyrosines at position 10 and 14.

Human CCR5 can be sulphated on tyrosine residues at positions 3, 10, 14 and 15, and sulphated residues contribute to the receptor binding of HIV [31]. Since residue 10 and 14 are within the core target linear epitope, we synthesised a synthetic N-terminal peptide which was sulphated at positions 10 and 14. Interestingly, RoAb13 bound more strongly (heteroclytic binding) to sulphated than non-sulphated forms of the peptide (Fig 5). The sera of the mice immunised with the synthetic peptide chimera were very heterogeneous with respect to binding to sulphated peptide. Mice 3 and 4, for example, failed to bind to the sulphated peptides, although binding strongly to the unsulfated form.

### Species specificity of the anti-CCR5 antibodies

**Fig 6:**
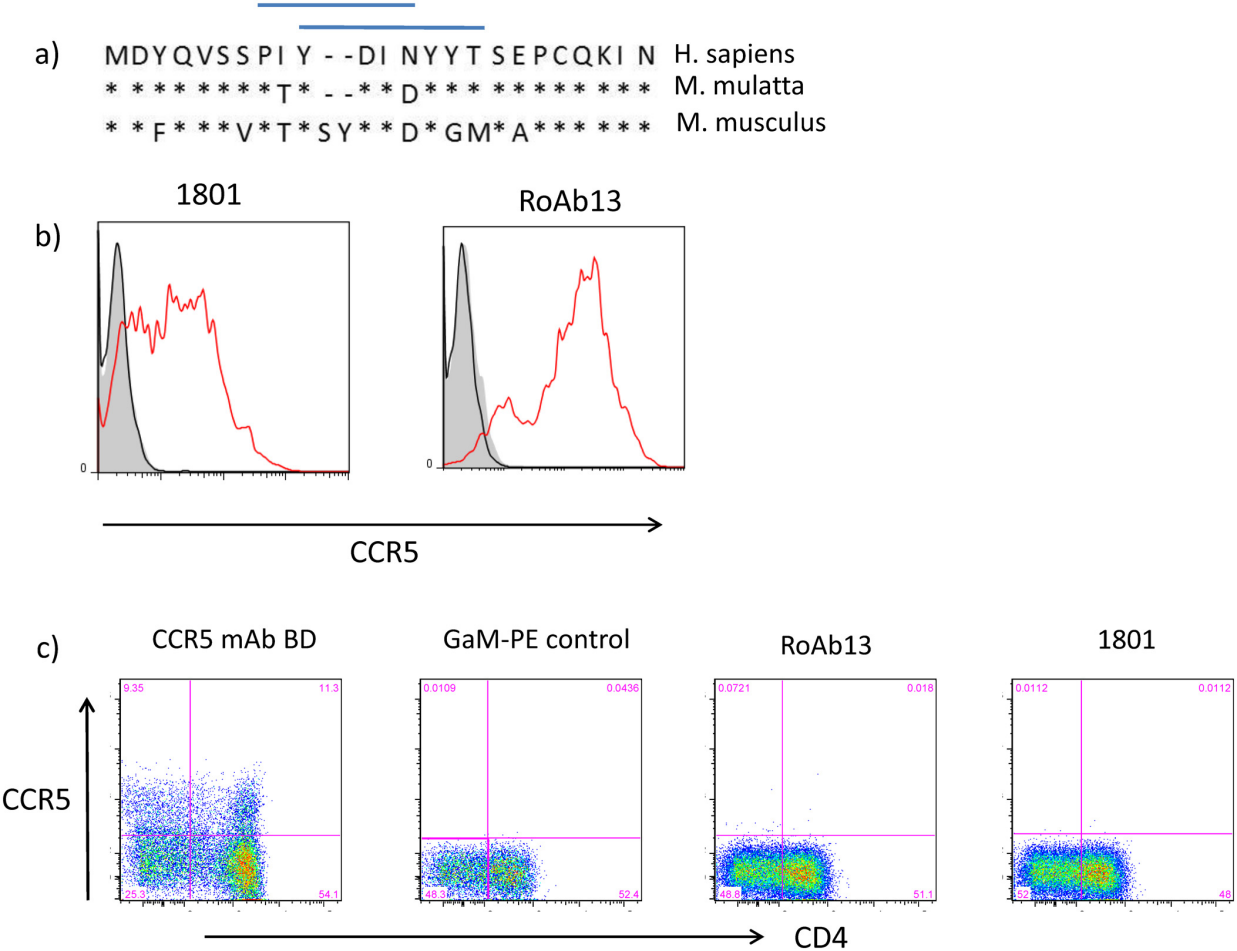
Species specificity of RoAb13 and R&D1801. A, The sequence of the N-terminal extracellular domain of CCR5 from human, rhesus Macaque or mouse CCR5. * identical amino acids—insertion in mouse sequence. B, Flow cytometry showing binding of R&D1801 or RoAb13 to CHO cells transfected with mouse (filled) or human (red line) CCR5. C, Flow cytometry showing binding of R&D1801 or RoAb13 or control to PBMC from rhesus macaques (indirect immunofluorescence, using Goat anti-mouse PE conjugate as second layer). The first panel shows binding of a commercially available PE-conjugated anti-rhesus CCR5 antibody (positive control).

The species specificity of the anti-CCR5 antibodies is important both for the purpose of designing functional experiments, and also in identifying key epitope positions required for antibody binding. The N-terminal portion of CCR5 of human, rhesus macaque and mouse are shown in Fig 6a. The mouse sequence contains a two amino acid insertion as well as mutations at both isoleucine and asparagine within the minimum linear epitope identified in Fig 2, and it was therefore unsurprising that there was no cross reaction with mouse CCR5 (Fig 6b). However, neither antibody showed any cross reaction with macaque CCR5, suggesting that the single change of asparagine to aspartic acid at position 13 abolished reactivity.

### RoAb13 binding blocks the chemotactic function of CCR5

**Fig 7:**
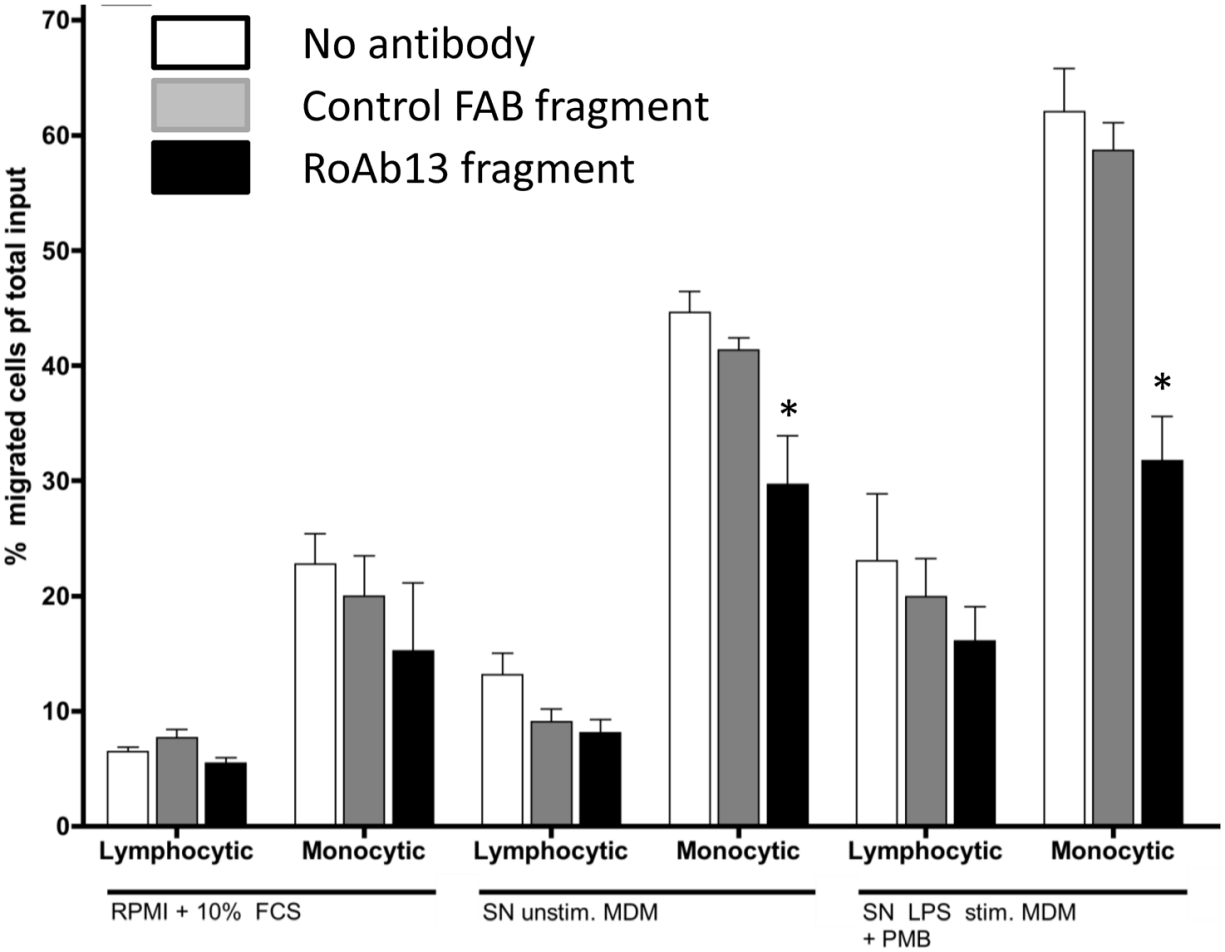
RoAb13 inhibits monocyte chemotaxis. PBMC were incubated in the upper chamber of transwells, while the lower chamber contained medium alone, supernatant of unactivated macrophages, or supernatant of macrophages activated with LPS (see M&M for details). RoAb13 Fab fragment or a control Fab fragement were added to some wells as shown. Migrating cells were collected from the lower chamber after three hours and the cells phenotyped as shown in S2 fig. Migration is shown as the proportion of cells in the PBMC premigration sample which migrate to the lower chamber during the assay. Each experimental condition was set up in triplet and * shows significant difference from control (T test, p<0.05).

Since we had access to the hybridoma producing RoAb13, we focused on this antibody and investigated its functional properties further. Previous studies had already shown that this antibody is a potent inhibitor of HIV infection [19,32]. However, the physiological function of CCR5 is to mediate chemokine-induced leukocyte migration. RoAb13 detected CCR5 on a subpopulation of human peripheral CD4, CD8 and NK cells Supporting Information S2 Fig). Monocytes and B cells showed low levels of CCR5 expression as detected with this antibody. The epitope of CCR5 targeted by RoAb 13 is therefore expressed on all major populations of CCR5 expressing cells. Addition of Fab fragment of RoAb13 to cultures of peripheral blood cells significantly inhibited the migration of monocytes, but not lymphocytes, in response to conditioned medium from both resting and activated macrophages (Fig 7, Supporting Information S3 Fig). The antibody also blocked migration in response to CCL2, CCL3 and CCL4, all of which are known to bind to CCR5, but failed to block migration in response to CXCL12 which acts via CXCR4 (Supporting Information S3 Fig). Similar results were obtained using whole RoAb13 antibody. These results support the hypothesis that the linear epitope of CCR5 is part of, or stearically close to the binding region of chemokine ligands.

### The crystal structure of RoAb13

**Fig 8:**
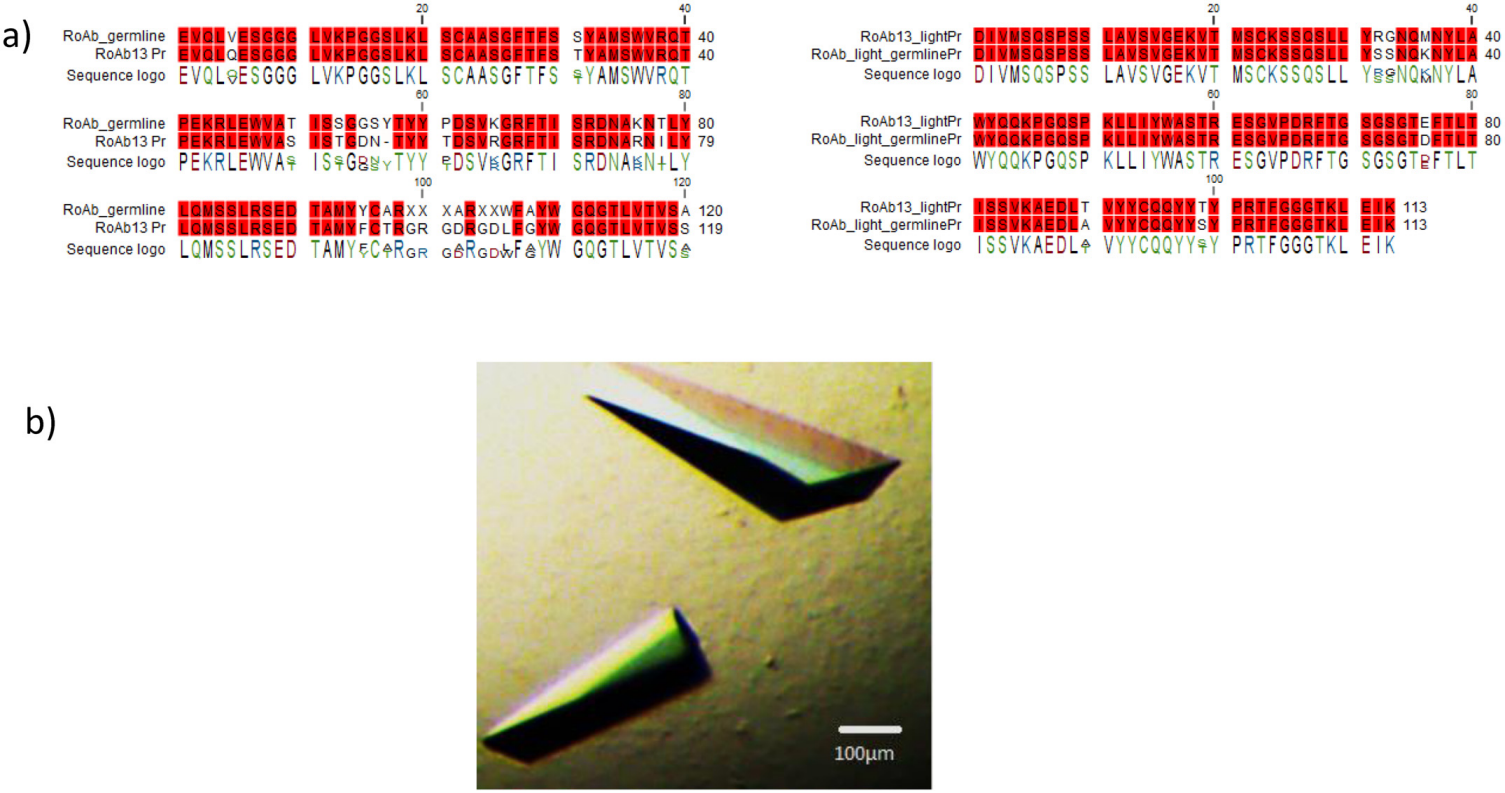
The sequence and crystals of RoAb13. A, The protein sequence of RoAb13 (lower sequence) compared to the closest germline sequence for heavy (right) and light (left) chains (as determined by the V-Quest). Conserved residues are shown in red. B, Crystals of RoAb13.

**Fig 9:**
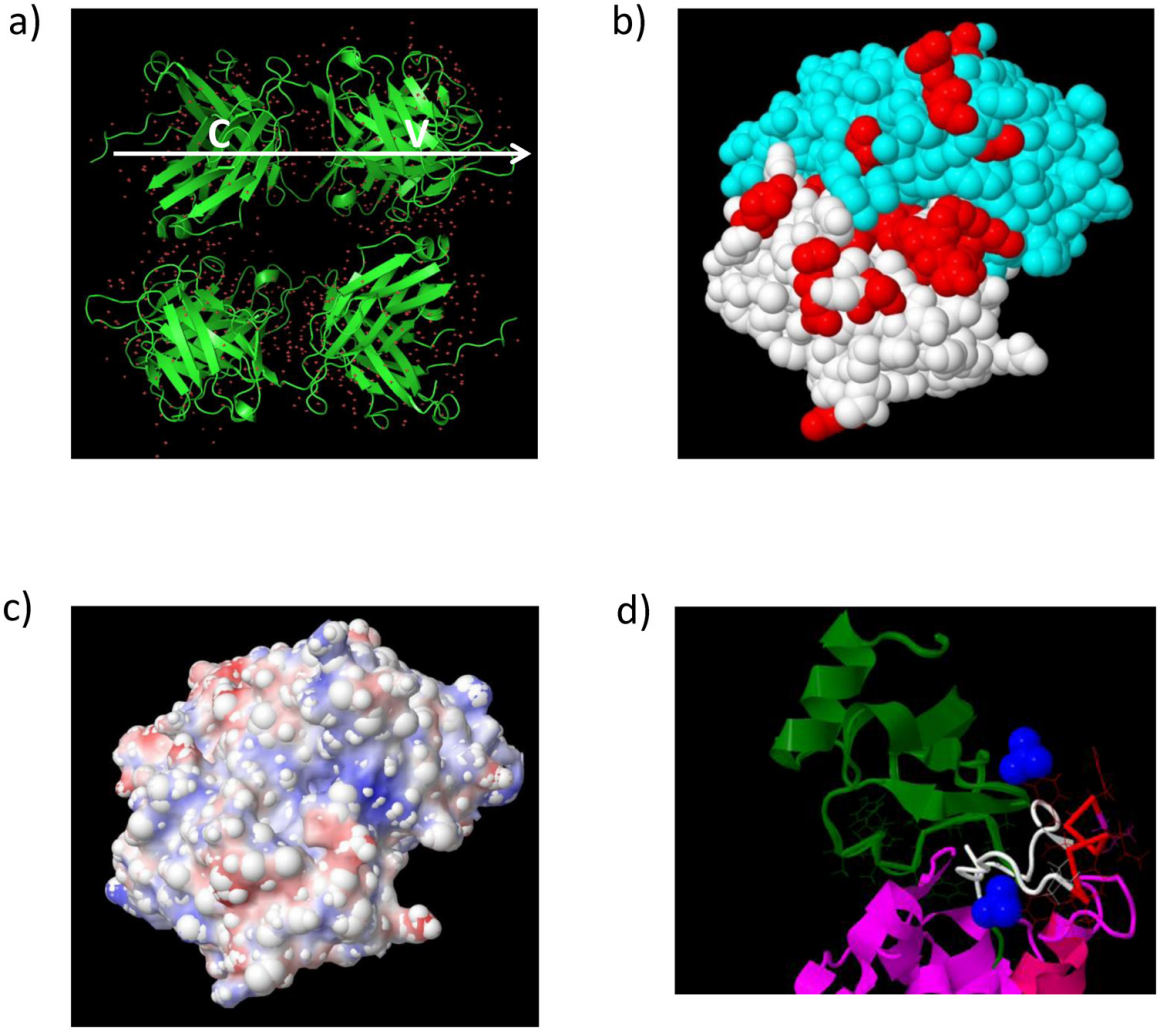
The crystal structure of RoAb13. A, The full structure of the RoAb13 Fab fragment showing two dimers in the asymmetric unit plus waters showing the position of the constant (C) and variable (V) domains for the upper molecule. B, A space filling model of RoAb13 structure. The molecule is oriented with the membrane distal surface (the antigen binding surface) upwards (the model shown in panel a is rotated so the white arrow shown is pointing upwards perpendicular to the page). The position of the amino acids which differ between germline and RoAb13 are shown in red. C, The electrostatic antigen binding surface of RoAb13. The orientation is the same as shown in b). Negatively charged areas are shown in red, while positively charged areas are shown in blue. D, The structure of CCR5 bound to its ligand, CCL5. The model is based on the pdb file (ccl5_ccr5.10.pdb) containing the coordinates from the optimum MD simulation described in [39], displayed and edited in Jmol. CCL5 is shown in green. The N-terminal domain of CCR5 is shown in white, except for the linear epitope 8–16, which is shown in red. The two tyrosine sulphate groups are shown in blue (spacefill).

Despite several attempts, we were unable to co-crystallise RoAb13 with peptides coding the linear epitope recognised by the antibody. We were, however, able to obtain high quality crystals of the RoAb13 Fab fragment itself. The crystals diffracting to 2.1 Å were obtained in the vapour diffusion experiments with 20% (w/v) PEG2K MME, 0.01M Nickel Chloride and 0.1M Tris pH 7.5 after 7 days. Fig 8 shows the RoAb13 sequence and crystals. The 3D structure is detailed in Fig 9. The crystallographic data and refinement details are shown in Supporting Information S2 Table. A comparison of the RoAb13 heavy and light chain amino acid sequence with the closest germ line sequences (Fig 8a) shows that the antibody (especially the heavy chain) has been modified by somatic cell hypermutation in several places, indicative of antigen-driven selection. The position of non-germline residues (including both hypermutated residues and non-templated amino acids introduced by junctional diversity) across the putative distal binding region of the antibody is shown in fig 9b. The distribution of the selected amino acids suggest that the linear epitope may adopt an extended conformation across the face of the antibody, with potential contact residues in both heavy and light chain. The electrostatic surface charge of RoAb13 in the same region is shown in Fig 9c. The existence of quite an extended area of positively charged (blue) surface may explain the enhanced binding to sulphated peptide Fig 5.

## Discussion

In this study, we have characterised two monoclonal antibodies against CCR5 which recognise a short linear epitope in the N-terminal region of the receptor. Our motivation is to understand the molecular details guiding this interaction, in order to develop novel antiviral or immunomodulatory therapeutic therapies. Therapeutic antibodies provide unsurpassed target specificity, and are increasingly finding a role in treatment of a broad spectrum of diseases. Antibody therapy for non-infectious diseases mostly targets unmodified human proteins, which are therefore autoantigens. It’s use in the clinic has been largely restricted to passive immunisation, via delivery of a preformed antibody produced ex vivo by cell culture. Active immunisation, in which patients produce their own antibodies in response to administration of an antigen is one of the most effective and cost effective medical interventions, preventing many important infectious diseases. In the context of autoantigens such as CCR5, however, the selection of a suitable immunogen becomes much more complex. The immunogen must bypass natural regulatory mechanisms which exist to prevent pathological autoimmunity, but at the same time it must avoid stimulating a cellular autoimmune response, since this is likely to lead to target cell death and hence toxicity. Both these considerations have prompted the investigation of short synthetic peptides as potential antibody immunogens. By coupling these peptides to non-self carriers (either peptides, or proteins), such immunogens can bypass the self-regulatory mechanisms and drive an auto-antigen response [33]. Furthermore, by judicious choice of peptide, it is possible to ensure that the self-peptide does not bind to MHC molecules, and hence will fail to drive an autoimmune cellular response. However, although this strategy has been explored for several decades, synthetic peptide immunogens remain an elusive goal. The principal reason for this is the exquisite conformational sensitivity of antibody, which means that immunogens must closely mimic the conformation of the antibody target if they are to drive effective antibody responses in vivo. The ability to mimic the three dimensional surface of a protein target (for example the chemokine/HIV binding surface of CCR5) with a synthetic peptide (or indeed any other synthetic structure) has provide to be extremely difficult. Linear epitopes (those created by a single contiguous stretch of amino acids on a protein surface) are attractive targets in this regard, since synthetic peptides coding such regions are at least in theory able to assume the required conformation, and thus mimic the intact protein molecule[34].

A large number of antibodies against CCR5 have been described, and several of these show potent ability to block HIV infection in vitro. However, the majority of antibodies have been generated using native CCR5 and recognise conformational epitopes on the extracellular loops of the receptor (e.g. [18,19]). In some cases, linear mimotopes which mimic such conformational epitopes [35] have been described. The existence of native linear epitopes have been inferred from the fact that some antibodies which bind to the N-terminal domain of CCR5 have been reported to bind CCR5 in Western blot, under conditions where the protein is largely denatured by the presence of detergents. Testing these antibodies on a synthetic peptide coding the entire extracellular region identified two independently generated antibodies which were shown to recognise distinct, but overlapping epitopeslying between amino acids 8 and16. The lack of binding of two other antibodies which also recognised CCR5 in Western blot may suggest that the protein does retain some three dimensional structure even under denaturing conditions, or alternatively that these antibodies require some post-translation modification in order to bind CCR5.

We characterised one antibody, RoAb13, in detail in order to understand its interaction with the linear epitope and hence guide strategies to generate antibody responses to this region of the receptor by passive immunisation with appropriate peptide analogues. This antibody bound to both linear peptide and native forms of the epitope. Interestingly binding to peptide was significantly enhanced by the sulphation of target tyrosines. Furthermore, binding of RoAb13 to CCR5 transfectants was quite heterogenous (see Fig 4c), also suggestive there may be different isoforms of the receptor[18]. Sulphation of CCR5 N-terminaltyrosines is known to occur in vivo[31], and indeed plays a crucial role in facilitating binding of both chemokine ligands[36] and HIV gp120 protein. The interaction between RoAb13 and CCR5 may be similar, in that the sulfate on one or more tyrosines may also contribute to stabilising RoAb13/CCR5 binding.

RoAb13 has previously been reported to inhibit HIV infection, in both single round and spreading infection assays [19]. This antibody showed only partial ability to block binding of CCL3, 4 and CCL5 to CHO-CCR5 transfected cells, or to block chemokine induced calcium flux in these cells. However, we found that RoAb13 effectively blocked chemokine induced migration of human peripheral blood leukocytes in responses to either individual chemokines or inflammatory macrophage conditioned media which is known to contain an array of chemokines. The clear cut inhibitory effects of the antibody even when using a cell supernatant containing a large mixture of inflammatory mediators, despite the well known redundancy in the chemokine network, suggest that CCR5 plays an important and non-redundant role in stimulating monocyte recruitment, and provides support for testing this antibody, or other anti-CCR5 monoclonals as a therapeutic intervention to block monocyte recruitment and hence dampen chronic inflammation [3][2].

Immunisation with a peptide which encoded the linear epitope recognised by RoAb13 together with a well-studied MHC promiscuous Tetanus Toxoid CD4 T helper cell epitope stimulated an antibody response in mice which cross-reacted with native CCR5, supporting the hypothesis that linear short epitopes could mimic certain stretches of the native CCR5. The response was quite heterogenous, both in quantity and quality. In part, this may reflect the fact the mice were outbred, and hence genetically heterogenous. However, the relatively low titre of antibody recognising native CCR5 compared to the titre of antibody which recognised a synthetic peptide encoding the epitope suggest that the immunogen is still not optimal. In part, this may reflect the lack of sulfation of the tyrosines within the B cell epitope of the immunising peptide. However, conformational considerations may still play an important role. A region within the extracellular domain adopts a helical conformation in solution[20], but NMR studies suggest it exists in a flexible state, and will adopt many configurations. In the context of the intact CCR5 the conformation of this stretch of amino acids is likely to be constrained. Furthermore, within the overall CCR5 structure, only one face of the peptide will be available for antibody binding. A crystal structure of CCR5 has recently been published [37]. However, the N-terminal amino acids encoding the linear epitope we define are missing, apparently because this region of the molecule was more dynamic and hence not well defined even within the crystal. A number of molecular dynamic simulations of the whole CCR5 binding to chemokine or HIV ligands have been published [38][39]. The proposed structure of the N-terminal portion of CCR5 bound to CCL5 is shown in Fig 9d (based on [20]and [39]). The positions of the linear epitope and its two tyrosine sulphate groups suggests that antibodies which bind in such an orientation as to interact with the sulphate residues (such as RoAb13) will be effective inhibitors of CCR5 ligand binding.

We reasoned that since RoAb13 bound both native CCR5 and the N-terminal peptide, a crystal structure of the antibody-peptide complex might give some further clues about the conformation of this region of CCR5. Despite several attempts, we were not successful in co-crystallising antibody bound to peptide. We did however, obtain both the sequence and a high quality structure for the RoAb13 molecule itself. A comparison of the sequence of RoAb13 with the nearest germ line sequences in the database showed that the sequence contains a number of sites of somatic hypermutation, as well as several non-template amino acids. Most of this variation, which is indicative of immune selection, is found in the heavy chain, but mutations are found across the distal surface of the molecule, suggesting the peptide binds in an extended conformation across this surface. The presence of an extended area of positive charge in the vicinity of non-template amino acids is suggestive of a possible site of tyrosine sulphate binding, but targeted mutagenesis of the antibody will be required to confirm this hypothesis.

In conclusion, we have characterised two monoclonal antibodies against CCR5 and their cognate epitope, a linear sequence in the N-terminal domain of CCR5. One antibody to this domain, RoAb13 combines potent HIV blocking activity with the ability to block chemokine induced monocyte migration. The domain contains at least one tyrosine sulphate which plays a role in facilitating antibody binding. The sequence and structure of the antibody are now made available, which will facilitate targeted modification of the antibody, and allow in silico modification of the peptide. Future studies using conformationally constrained sulphated variants of this B cell epitope may provide a strategy to bypass autoimmunity and generate a therapeutic anti-CCR5 response by active immunisation.

## Supporting Information

S1 Fig Calibration beads for quantifying number of RoAb13 molecules bound to cells. (TIF)

S2 Fig Expression of CCR5 on human peripheral blood subsets as detected by RoAb13. (TIF)

S3 Fig RoAb13 inhibits migration induced by chemokines which bind CCR5. (TIF)

S4 Fig 12.5% SDS PAGE electrophoresis of RoAb13 before and after FAB digestion and purification. (TIF)

S1 Table Primers for RoAb13 cloning. (DOCX)

S2 Table Crystallographic data and refinement details. (DOCX)
