# Supplementary figures and images for: A Linear Epitope in the N-Terminal Domain of CCR5 and Its Interaction with Antibody
Source: PLoS One. 2015 Jun 1;10(6):e0128381. doi: 10.1371/journal.pone.0128381 (PMC4451072; doi:10.1371/journal.pone.0128381)

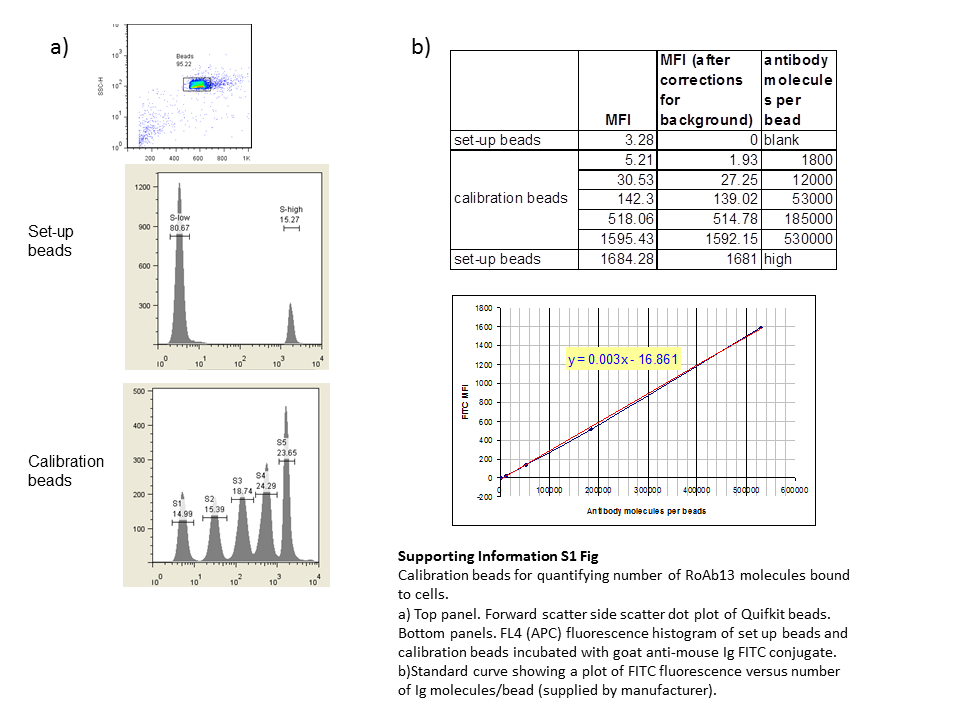

Supplement: S1 Fig — (TIF) [file pone.0128381.s001.TIF]

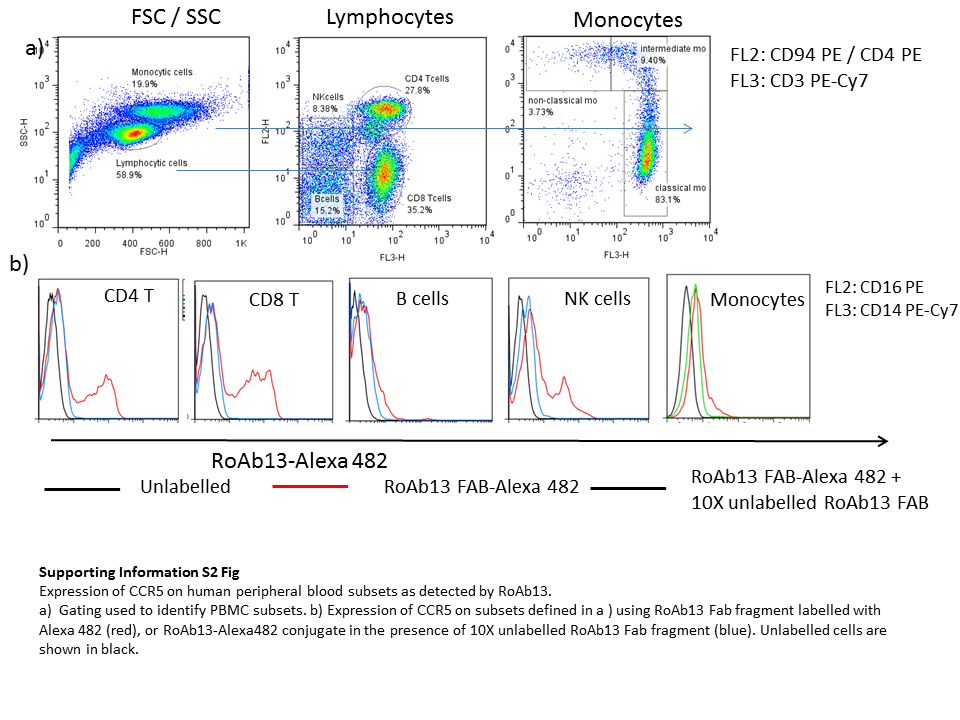

Supplement: S2 Fig — (TIF) [file pone.0128381.s002.TIF]

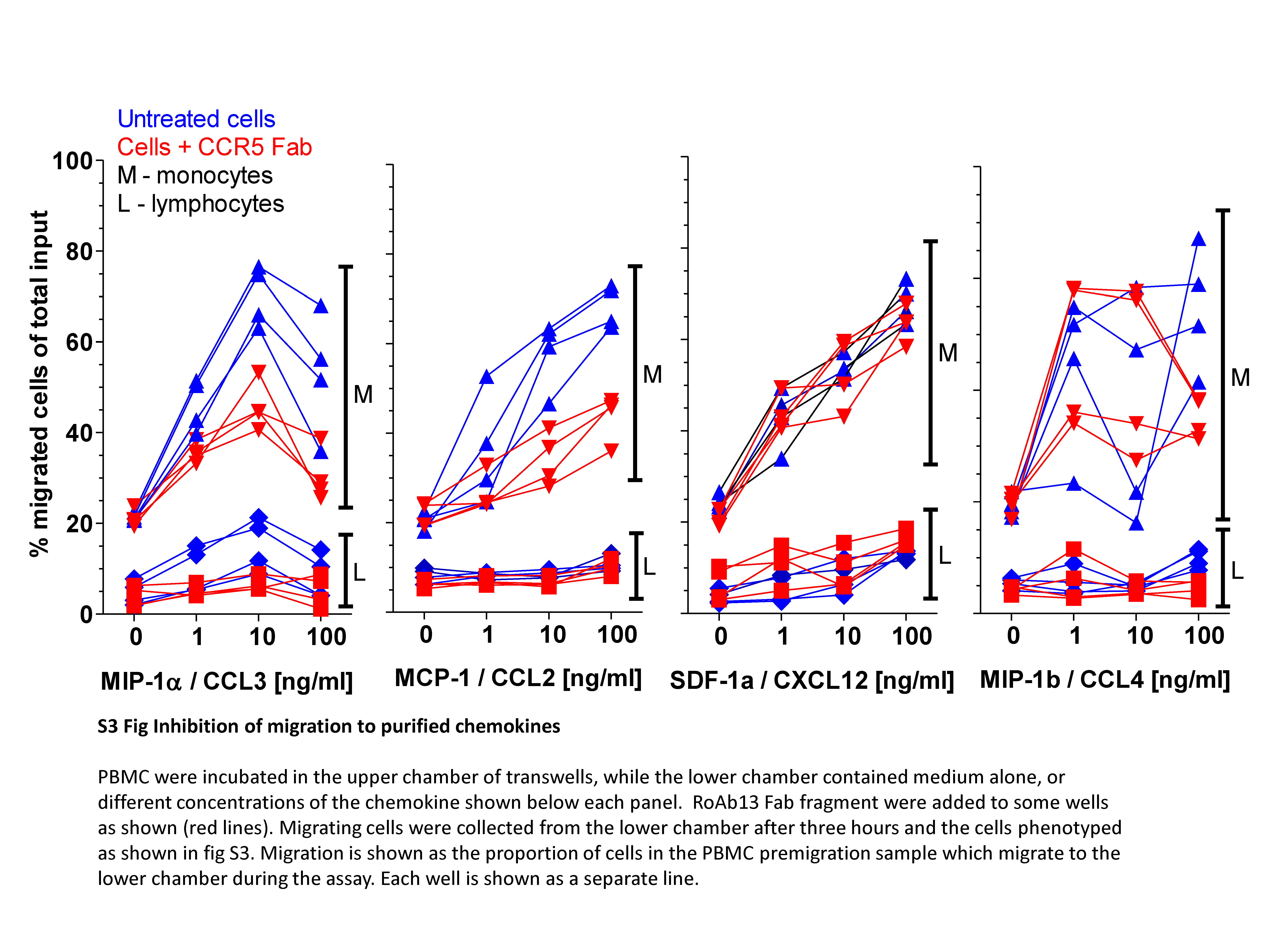

Supplement: S3 Fig — (TIF) [file pone.0128381.s003.TIF]

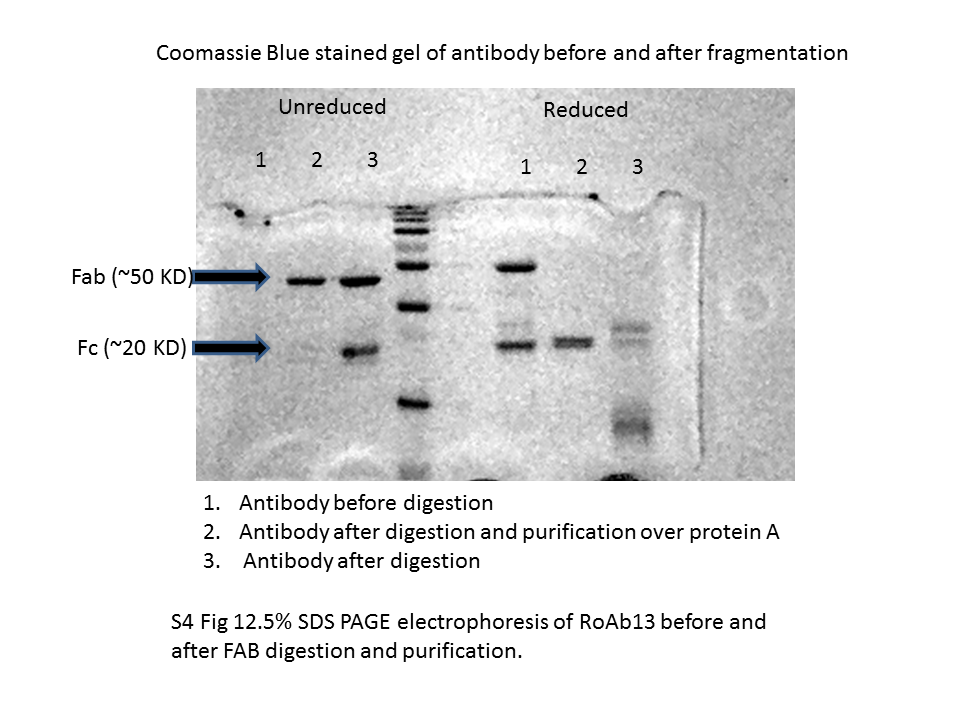

Supplement: S4 Fig — (TIF) [file pone.0128381.s004.TIF]
