# Supplementary material for: A Linear Epitope in the N-Terminal Domain of CCR5 and Its Interaction with Antibody
Source: PLoS One. 2015 Jun 1;10(6):e0128381. doi: 10.1371/journal.pone.0128381 (PMC4451072; doi:10.1371/journal.pone.0128381)
Supplement: S1 Table — (DOCX) [file pone.0128381.s005.docx]

**Table S1 Primers for RoAb13 cloning**

| IgG2aRC outer (for RT) | GTCCAGTGGTGTGCACACCTTCC |
| --- | --- |
| IgG2aRC inner (for RACE amplification) | GTCAAGGGTTATTTCCCTGAGC |
| IgKappaRC | AACTGTATCCATCTTCCCACCATC |
|  |  |
|  |  |
