# Supplementary material for: A Linear Epitope in the N-Terminal Domain of CCR5 and Its Interaction with Antibody
Source: PLoS One. 2015 Jun 1;10(6):e0128381. doi: 10.1371/journal.pone.0128381 (PMC4451072; doi:10.1371/journal.pone.0128381)
Supplement: S2 Table — (DOCX) [file pone.0128381.s006.docx]

**Table S2** Crystallographic data and refinement details.

| **Space group** | P2_1_ |
| --- | --- |
| **Cell** | a=75.20 Å  b=88.64 Å  c=78.45 Å  β = 103.19° |
| **Data collection** | |
| **Temperature (K)** | 100 |
| **Wavelength (Å)** | 1.5418 |
| **Resolution (Å)** | 76.4 – 2.1  (2.21- 2.10)* |
| **Redundancy** | 3.5 (3.3)* |
| **R_merge_ (%)** | 8 (44)* |
| **I/σ(Ι)** | 8.70 (1.45)* |
| **Calculated solvent content (%)** | 48.7 |
| **Refinement** | |
| **Refinement program** | Phenix.refine |
| **Resolution (Å)** | 15.40 - 2.10 |
| **Unique reflections** | 55811 |
| **Completeness (%)** | 95.5 |
| **R factor (%)** | 18.6 |
| **R_free_ (%)** | 23.4 |
| **rmsd from ideal bond lengths (Å)** | 0.008 |
| **rmsd from ideal angles (°)** | 1.2 |
| **Average B-factor for protein (Å^2­^)** | 28.0 |
| **Non-Gly/Pro residues in most favoured regions** | 88.9% (668/751) |
| **Non-Gly/Pro residues in additionally allowed regions** | 10.4% (78/751) |
| **Non-Gly/Pro residues in disallowed regions** | 0.7% (5/751) |

* values in parentheses are for the outermost shell

R_merge_ = Σ |I_h_ – <I_h_>|/ Σ I_h_

R-factor = Σ (|F_obs_| - |F_calc_|)/ Σ |F_obs_|
